# Development of a high-affinity anti-bovine PD-1 rabbit–bovine chimeric antibody using an efficient selection and large production system

**DOI:** 10.1186/s13567-023-01213-6

**Published:** 2023-09-27

**Authors:** Tomohiro Okagawa, Satoru Konnai, Shinya Goto, Yamato Sajiki, Otgontuya Ganbaatar, Kei Watari, Hayato Nakamura, Cai-Xia Wang, Taro Tachibana, Yukinari Kato, Yayoi Kameda, Junko Kohara, Nobuhiro Terasaki, Manabu Kubota, Akira Takeda, Hirofumi Takahashi, Yasuhiko Suzuki, Naoya Maekawa, Shiro Murata, Kazuhiko Ohashi

**Affiliations:** 1 Department of Advanced Pharmaceutics, Faculty of Veterinary Medicine, Hokkaido University, Sapporo, Japan; 2 Department of Disease Control, Faculty of Veterinary Medicine, Hokkaido University, Sapporo, Japan; 3 Institute for Vaccine Research and Development (HU-IVReD), Hokkaido University, Sapporo, Japan; 4 Department of Bioengineering, Graduate School of Engineering, Osaka City University, Osaka, Japan; 5 Department of Bioengineering, Graduate School of Engineering, Osaka Metropolitan University, Osaka, Japan; 6 Department of Antibody Drug Development, Tohoku University Graduate School of Medicine, Sendai, Japan; 7 Department of Molecular Pharmacology, Tohoku University Graduate School of Medicine, Sendai, Japan; 8 Division of Bioresources, International Institute for Zoonosis Control, Hokkaido University, Sapporo, Japan; 9 Animal Research Center, Agriculture Research Department, Hokkaido Research Organization, Shintoku, Japan; 10 Hokkaido Agricultural Mutual Aid Association, Shibecha, Japan; 11 Global Station for Zoonosis Control, Global Institution for Collaborative Research and Education (GI-CoRE), Hokkaido University, Sapporo, Japan; 12 International Affairs Office, Faculty of Veterinary Medicine, Hokkaido University, Sapporo, Japan

**Keywords:** Monoclonal antibodies, programmed death-1, cattle, antibody production

## Abstract

Immune checkpoint molecules PD-1/PD-L1 cause T-cell exhaustion and contribute to disease progression in chronic infections of cattle. We established monoclonal antibodies (mAbs) that specifically inhibit the binding of bovine PD-1/PD-L1; however, conventional anti-PD-1 mAbs are not suitable as therapeutic agents because of their low binding affinity to antigen. In addition, their sensitivity for the detection of bovine PD-1 is low and their use for immunostaining PD-1 is limited. To address these issues, we established two anti-bovine PD-1 rabbit mAbs (1F10F1 and 4F5F2) and its chimeric form using bovine IgG1 (Boch1D10F1), which exhibit high binding affinity. One of the rabbit mAb 1D10F1 binds more strongly to bovine PD-1 compared with a conventional anti-PD-1 mAb (5D2) and exhibits marked inhibitory activity on the PD-1/PD-L1 interaction. In addition, PD-1 expression in bovine T cells could be detected with higher sensitivity by flow cytometry using 1D10F1. Furthermore, we established higher-producing cells of Boch1D10F1 and succeeded in the mass production of Boch1D10F1. Boch1D10F1 exhibited a similar binding affinity to bovine PD-1 and the inhibitory activity on PD-1/PD-L1 binding compared with 1D10F1. The immune activation by Boch1D10F1 was also confirmed by the enhancement of IFN-γ production. Finally, Boch1D10F1 was administered to bovine leukemia virus-infected cows to determine its antiviral effect. In conclusion, the high-affinity anti-PD-1 antibody developed in this study represents a powerful tool for detecting and inhibiting bovine PD-1 and is a candidate for PD-1-targeted immunotherapy in cattle.

**Supplementary Information:**

The online version contains supplementary material available at 10.1186/s13567-023-01213-6.

## Introduction

Programmed death-1 (PD-1) is an immune checkpoint molecule that interacts with its ligand PD-ligand 1 (PD-L1) to cause the functional exhaustion of T cells, which is evident through the loss of effector function by inhibiting T-cell receptor (TCR) signaling [1–3]. During chronic infection, PD-1 is upregulated on T cells as a result of persistent stimulation via TCR, co-receptors, and cytokines [4]. In cattle, the functional exhaustion of T cells is caused by the upregulation of the PD-1/PD-L1 pathway and results in disease progression during chronic infections, such as bovine leukemia virus (BLV) infection [5–8]. BLV is a retrovirus which infects bovine B cells and develops into a lifelong infection in cattle. It can also result in an aggressive lymphoma, known as enzootic bovine leukosis (EBL), in 1–5% of the infected cattle after a long latent period [9]. In previous studies, we developed anti-bovine PD-1 and anti-bovine PD-L1 rat-bovine chimeric antibodies (chAbs) in a mammalian expression system and tested their antiviral effects in clinical studies using BLV-infected cattle [10–12]. Blockade of the binding of PD-1/PD-L1 by these chAbs restored T-cell function and exerted antiviral effects in the BLV-infected cattle [11, 12].

Although the antiviral effect was observed by treatment with anti-bovine PD-1 rat-bovine chAb (Boch5D2), the binding affinity of Boch5D2 to bovine PD-1 was relatively low compared with that of commercial anti-human PD-1 blocking antibodies for immunotherapy [13, 14]. Recently, novel therapeutic antibodies with higher binding affinities to PD-1 have been developed to enhance the clinical benefit of immunotherapy in humans [13, 14]. In addition, the application of immunostaining for flow cytometry using anti-bovine PD-1 rat mAb (5D2) established in a previous study [7] was also limited because of its low binding affinity [10]. Indeed, PD-1 expression was detectable by flow cytometry using 5D2 only in T cells of the cattle experiencing a robust stimulation by antigens during chronic infection [8, 15–17]. To address the mechanism of T-cell exhaustion mediated by PD-1 in various bovine diseases, a novel mAb capable of detecting bovine PD-1 with high sensitivity in the flow cytometric analysis is required.

Mice and rats are widely used for immunization to generate mAbs; however, the use of rabbits for the mAb development has attracting interest because of their unique and highly distinctive antibody repertoire, which exhibits high-affinity, specificity, and diversity [18]. Indeed, previous studies have been succeeded in developing high-affinity mAbs to several antigens using rabbits [19–21]. These findings prompted us to establish a rabbit mAb against bovine PD-1.

Gram-scale quantities of antibody are required for clinical trials of therapeutic antibodies in adult cattle. We have established an expression system for large amounts of antibody using a mammalian cell line and an engineered expression plasmid with a DHFR marker for efficient selection of transfected cells and a ubiquitous chromatin opening element (UCOE) to reduce DNA methylation in the transgene promoter [22, 23]. We applied this system for the large production of chAb for a clinical trial in cattle.

In this study, we generated an anti-bovine PD-1 rabbit mAb (1D10F1) and rabbit–bovine chAb (Boch1D10F1) which exhibit high-affinity to bovine PD-1. The biochemical properties of these antibodies were compared with anti-bovine PD-1 rat mAb (5D2) and chAb (Boch5D2) which were established previously [7, 10]. Accordingly, mass production of Boch1D10F1 was achieved and administration of Boch1D10F1 to BLV-infected cattle was conducted to evaluate the antiviral effects of this high-affinity antibody.

## Materials and methods

### Generation of anti-bovine PD-1 rabbit mAbs

A rabbit was immunized with recombinant bovine PD-1-Ig fusion protein [24] in TiterMax Gold Adjuvant (Sigma–Aldrich, St. Louis, MO, USA). Lymphocytes were collected from the immunized rabbit 3 weeks later, were fused with SP2 myeloma cells. Culture supernatants from heterohybridomas [25, 26] were screened using ELISA with recombinant polyhistidine-tagged bovine PD-1 protein (BoPD-1-His) [10] and horseradish peroxidase (HRP)-conjugated anti-rabbit IgG (H + L) antibody (Jackson ImmunoResearch, West Grove, PA, USA). The hybridomas that tested positive in ELISA were cloned by limiting dilution. Culture supernatant of cultivated clones were tested again by ELISA against BoPD-1-His and total RNA was isolated from the positive clones of hybridomas using TRIzol reagent (Thermo Fisher Scientific, Waltham, MA, USA) according to the manufacturer’s instructions. cDNAs were synthesized from the RNA samples with SuperScript III One-Step RT-PCR System with Platinum Taq High Fidelity DNA Polymerase (Thermo Fisher Scientific). The gene encoding variable regions of heavy and light chains of antibody (VH and VL) were amplified using KOD -Plus- Neo DNA Polymerase (Toyobo, Osaka, Japan) and gene specific primers. The amplicons were cloned into pCAGGS [27] with the nucleotides encoding constant regions of heavy and light chains (CH and CL) of rabbit IgG and sequenced by Eurofins Genomics (Tokyo, Japan). HEK293T cells were transfected with the expression plasmids and grown in fetal bovine serum (FBS)-free D-MEM medium (Fujifilm Wako Pure Chemical, Osaka, Japan). The culture supernatants were tested by ELISA against bovine PD-1 as shown above. The supernatants from two clones (1D10F1 and 4F5F2) were positive in ELISA and further tested in the following screening test using purified antibodies.

### Expression and purification of anti-bovine PD-1 rabbit mAbs

Transient cell lines expressing anti-bovine PD-1 rabbit mAbs were established using the Expi293 Expression System (Thermo Fisher Scientific). Briefly, Expi293F cells were transfected with the expression plasmids, pCAGGS-1D10F1-VHCH and pCAGGS-1D10F1-VLCL or pCAGGS-4F5F2-VHCH and pCAGGS-4F5F2-VLCL, using Expifectamine (Thermo Fisher Scientific) and cultured with shaking in Expi293 Medium (Thermo Fisher Scientific) at 37 °C and 125 rpm with 8% CO_2_ for 7 days. For negative control antibodies, cells were transfected with a combination of pCAGGS-1D10F1-VHCH and pCAGGS-4F5F2-VLCL (1H4L) or the other combination (4H1L). Antibodies were purified from the culture supernatants by affinity chromatography with an Ab-Capcher ExTra (ProteNova) and the buffer was exchanged with phosphate-buffered saline (PBS) by size exclusion chromatography using PD-10 Desalting Column (GE Healthcare). The concentration of Boch5D2 was measured by ultraviolet (UV) absorbance at 280 nm with a NanoDrop 8000 spectrophotometer (Thermo Fisher Scientific).

### Expression and purification of anti-bovine PD-1 bovine-rabbit chAb

The nucleotide sequences of the VH and VL genes of 1D10F1 were combined with the constant regions of bovine IgG_1_ (GenBank accession number X62916) with reduced Fc-mediated effector functions (IgG_1_ ADCC −) [10] and bovine Ig lambda (GenBank accession number X62917), respectively. In selecting the subclasses of the constant region, the dominant subclasses in bovine immunoglobulins (IgG1 and Igλ) were chosen [28, 29]. The resulting sequences were modified according to the optimal codon usage of the Chinese hamster, synthesized (GenScript), and cloned into the expression vectors, pDC62c5-U533, pDC61, and pNC32c5-U533 [23].

CHO DG44 cells (a *dhfr*-deficient CHO cell line) were transfected with the expression vectors and cloned by limiting dilution in CD OptiCHO medium (Thermo Fisher Scientific) supplemented with 2 mM GlutaMAX-I (Thermo Fisher Scientific). For the selection of cells transfected with pNC32c5-U533, 800 μg/mL of G418 sulfate (Enzo Life Sciences) were added to the medium. After 3 weeks, the cloned cells were screened for their ability to produce Boch1D10F1 using an enzyme-linked immunosorbent assay (ELISA) with horseradish peroxidase (HRP)-conjugated anti-bovine IgG Fc rabbit polyclonal antibody (Rockland Immunochemicals) as previously described [24]. Boch1D10F1 was produced by shaking the established cell lines in Dynamis Medium (Thermo Fisher Scientific) with EfficientFeed B + AGT Supplement (Thermo Fisher Scientific) at 37 °C with 5% CO_2_ for 14 days. Live and dead cells were counted with a Countess Automated Cell Counter (Thermo Fisher Scientific). The concentration of Boch1D10F1 in the culture supernatant was determined using a bovine IgG ELISA as described above.

Purification of Boch1D10F1 from the culture supernatant was performed by affinity chromatography with an Ab-Capcher ExTra (ProteNova) and PD-10 Desalting Column (GE Healthcare) as described above. The concentration of the purified Boch1D10F1 was measured by UV absorbance at 280 nm with a NanoDrop 8000 spectrophotometer (Thermo Fisher Scientific). The purity of Boch1D10F1 was confirmed by sodium dodecyl sulfate–polyacrylamide gel electrophoresis (SDS-PAGE) under reducing or nonreducing conditions on a SuperSep Ace 5%–20% gradient polyacrylamide gel (Fujifilm Wako Pure Chemical) with 2 × Laemmli Sample Buffer (Bio-Rad, Hercules, CA, USA). The Precision Plus Protein Dual Standard (Bio-Rad) was used as a molecular weight size marker and the proteins were visualized with Quick-CBB (Fujifilm Wako Pure Chemical).

### Binding assay of anti-bovine PD-1 antibodies

To confirm the binding activity of the anti-bovine PD-1 rabbit mAbs and chAb (Boch1D10F1) to bovine PD-1 protein, flow cytometry was performed using myc-tagged bovine PD-1-expressing CHO DG44 cells (BoPD-1-myc cells) [7]. Briefly, BoPD-1-myc cells were incubated with the purified mAbs (1D10F1, 4F5F2, 1H4L, or 4H1L), anti-bovine PD-1 rat mAb (5D2) [7], rat IgG_2a_ isotype control (R35-95, BD Biosciences), Boch1D10F1, or bovine IgG control (Sigma–Aldrich) at room temperature for 30 min. The cells were then washed with PBS supplemented with 1% bovine serum albumin (BSA; Sigma–Aldrich) and labeled with Alexa Flour 647-conjugated anti-rabbit IgG (H + L) goat F(ab')2 (Thermo Fisher Scientific), APC-conjugated anti-rat immunoglobulin antibody (Southern Biotech) or APC-conjugated anti-bovine IgG Fc goat antibody (Jackson ImmunoResearch) at room temperature for 30 min. Finally, the cells were washed and analyzed immediately using FACS Verse (BD Biosciences).

To evaluate the detection sensitivity and specificity of anti-bovine PD-1 rabbit mAbs to PD-1 protein in bovine lymphocytes, multicolor flow cytometric analysis was performed using bovine leukocytes isolated from fresh blood samples. To prevent nonspecific reactions of staining antibodies, leukocytes were incubated in PBS containing 10% goat serum (Sigma–Aldrich) at room temperature for 15 min. The cells were stained with anti-bovine PD-1 rabbit mAb (1D10F1 or 4F5F2), rabbit IgG controls (1H4L), anti-bovine PD-1 rat mAb (5D2) [7], or rat IgG_2a_ isotype control (R35-95, BD Biosciences) for 30 min at room temperature. After washing with 1% BSA-PBS, the cells were labeled with Alexa Flour 647-conjugated anti-rabbit IgG (H + L) goat F(ab')2 (Thermo Fisher Scientific) or APC-conjugated anti-rat immunoglobulin antibody (Southern Biotech) for 30 min at room temperature with a cocktail of staining antibodies, including PerCp/Cy5.5-conjugated anti-bovine CD3 mAb (MM1A; Washington State University Monoclonal Antibody Center), FITC-conjugated anti-bovine CD4 mAb (CC8; Bio-Rad), PE-conjugated anti-bovine CD8 mAb (CC63, Bio-Rad), APC/Cy7-conjugated anti-TCR1-N24 mAb (GB21A; Washington State University Monoclonal Antibody Center), and PE/Cy7-conjugated anti-bovine IgM mAb (IL-A30; Bio-Rad). The mAbs MM1A, GB21A, and IL-A30 were conjugated with each fluorochrome using Lightning-Link Conjugation Kits (Innova Biosciences). Finally, the cells were washed and analyzed by FACS Verse (BD Biosciences).

### Surface plasmon resonance analysis

To assess the binding affinity of 1D10F1, 4F5F2, and Boch1D10F1 to bovine PD-1, surface plasmon resonance (SPR) analysis was performed using the Biacore System (GE Healthcare) with BoPD-1-His as described previously [10]. SPR measurements were performed using a CM5 sensor chip (GE Healthcare) immobilized with BoPD-1-His and 1D10F1, 4F5F2, 5D2, and Boch1D10F1 on a Biacore X100 or Biacore 3000 instrument (GE Healthcare) at 25 °C. The kinetic constant of each antibody was determined by fitting with a 1:1 kinetic binding model.

### Blockade assay of PD-1/PD-L1 binding

To confirm the ability of 1D10F1, 4F5F2, and Boch1D10F1 to block PD-1/PD-L1 binding, biotinylated BoPD-1-Ig (5 μg/mL) [24] was incubated with various concentrations (0.39 to 50 μg/mL) of 1D10F1, 4F5F2, and Boch1D10F1 at 37 °C for 30 min. The incubated BoPD-1-Ig proteins were incubated with bovine PD-L1-EGFP-expressing CHO DG44 cells (BoPD-L1-EGFP cells) [24] at 37 °C for 30 min. BoPD-1-Ig bound to BoPD-L1-EGFP cells was labeled with APC-conjugated streptavidin (BioLegend) at room temperature for 30 min, washed with PBS, and analyzed by FACS Verse (BD Biosciences). Rat IgG_2a_ isotype control (R35-95, BD Biosciences), rabbit IgG controls (1H4L and 4H1L), and bovine IgG_1_ control (Bethyl Laboratories) were used as negative controls.

### Immune activation assay by Boch1D10F1

To analyze immune activation by Boch1D10F1, peripheral blood mononuclear cell (PBMC) cultivation assay was conducted as previously described with some modifications [8]. PBMCs were isolated from the peripheral blood of BLV-infected cattle by density gradient centrifugation using Percoll (GE Healthcare), washed three times with PBS, and suspended in RPMI1640 Medium (Sigma–Aldrich) supplemented with 10% heat-inactivated FBS (Thermo Fisher Scientific), 200 IU/mL of penicillin, 200 μg/mL of streptomycin, and 0.01% L-glutamine (Thermo Fisher Scientific). PBMCs were cultured with 20 μg/mL of Boch1D10F1, anti-bovine PD-1 rat-bovine chAb (Boch5D2) [10], or bovine IgG control (Sigma–Aldrich) in the presence of 2% heat-inactivated supernatant from BLV-infected fetal lamb kidney cells (FLK-BLV) at 37 °C in 5% CO_2_ for six days. Culture supernatants were then harvested and IFN-γ concentrations in the supernatants were determined using a bovine IFN-γ ELISA (Mabtech) performed in duplicate according to the manufacturer’s protocol.

### Evaluation of the antiviral effect of Boch1D10F1 treatment in BLV-infected cattle

To confirm the antiviral effects of Boch1D10F1 in cattle, the administration of Boch1D10F1 was performed in cattle naturally infected with BLV (*n* = 6, Holstein, female; Additional file 1) in animal facilities at the Animal Research Center, Agricultural Research Department, Hokkaido Research Organization (Shintoku, Hokkaido, Japan) and Faculty of Veterinary Medicine, Hokkaido University (Sapporo, Hokkaido, Japan). BLV infection was confirmed by the detection of anti-BLV antibodies in plasma using a commercial ELISA kit (JNC, Tokyo, Japan) and by the detection of BLV provirus in DNA using real-time PCR as described previously [10]. The number of lymphocytes in blood samples was counted using an automated hematology analyzer (Celltac α; Nihon Kohden, Tokyo, Japan). BLV-infected cattle were classified as aleukemic (AL) or persistent lymphocytosis (PL) based on the lymphocyte counts as follows: AL < 8000 cells/μL; PL > 8000 cells/μL.

Four BLV-infected cattle (AL2, AL3, PL2, and PL3) were intravenously administered 1 mg/kg (AL2 and AL3) or 2 mg/kg (PL2 and PL3) of purified Boch1D10F1 diluted in saline (Additional file 1). Two of the animals (AL3 and PL3) were also administrated 0.5 mg/kg of meloxicam (Metacam; Boehringer Ingelheim, Ingelheim, Germany) subcutaneously three times at seven-days intervals (Additional file 1). The other two animals (AL1 and PL1) did not receive any treatment and were used as negative controls (Additional file 1). This animal experiment was approved by the Ethics Committee of the Animal Research Center, Agricultural Research Department, Hokkaido Research Organization (approval #1703) and the Ethics Committee of the Faculty of Veterinary Medicine, Hokkaido University (Approval #17-0024).

To determine proviral loads in the peripheral blood, the BLV *tax* gene was measured in genomic DNA by quantitative real-time PCR using the Cycleave PCR Reaction Mix (Takara Bio) and a Probe/Primer Mix for BLV (Takara Bio) with a LightCycler 480 System II (Roche Diagnostics) as described previously [10]. Each DNA sample was tested in triplicate. Genomic DNA was extracted from PBMCs with the Wizard Genomic DNA Purification Kit (Promega) and the concentration of DNA was measured by UV absorbance at 260 nm with a NanoDrop 8000 spectrophotometer (Thermo Fisher Scientific).

### Statistical analysis

All statistical tests were performed with GraphPad Prism 6 software (GraphPad Software Inc.). Differences were considered statistically significant at *p* < 0.05.

## Results

### Establishment and characterization of anti-bovine PD-1 rabbit mAbs

**Figure 1:**
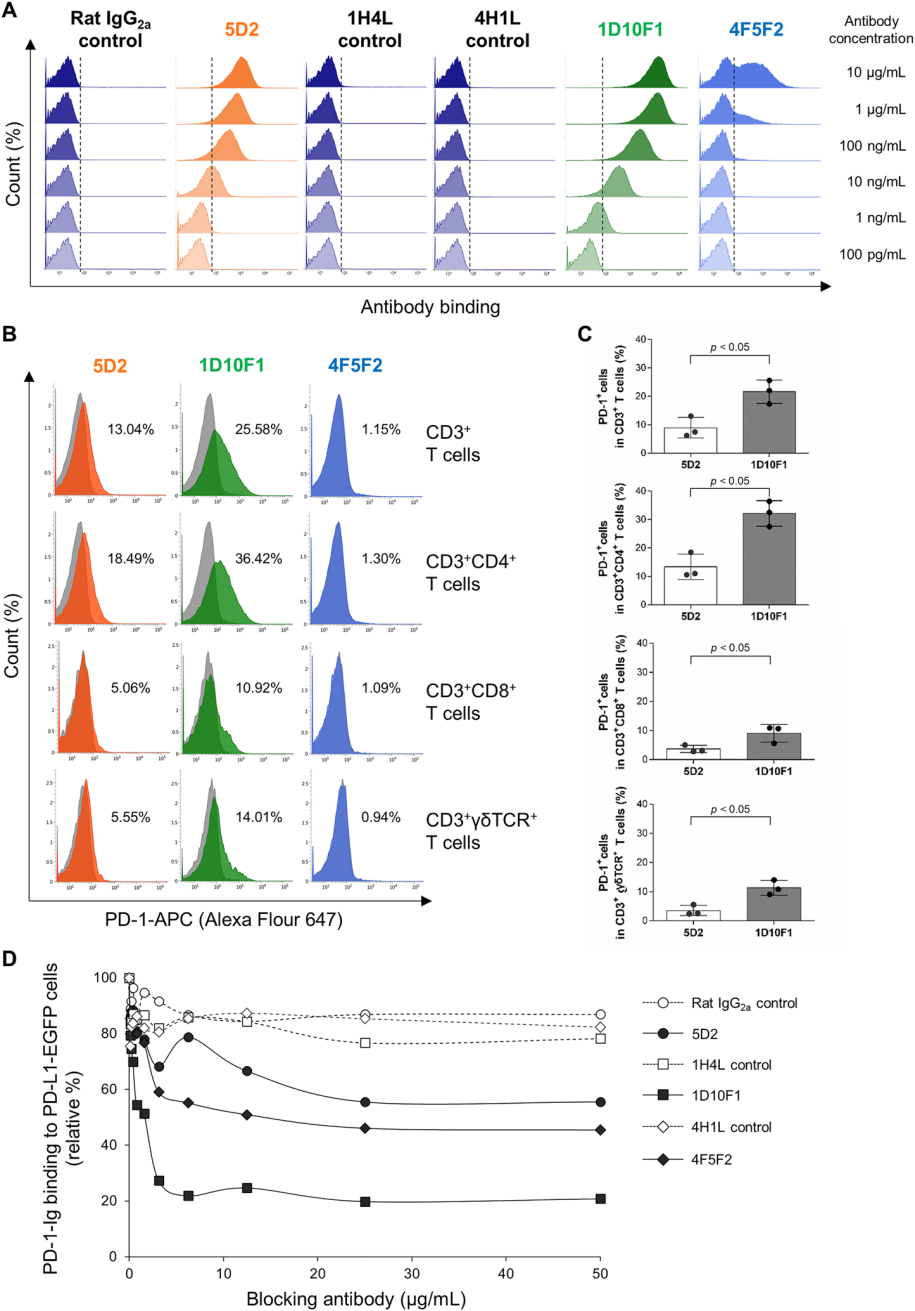
**Generation and characterization of anti-bovine PD-1 rabbit mAbs.** **A** Flow cytometric analysis using anti-bovine PD-1 rabbit mAbs (1D10F1 and 4F5F2) and anti-bovine PD-1 rat mAb (5D2). BoPD-1-myc cells were stained with the mAbs using serial dilutions (10 μg/mL to 100 pg/mL). Rat IgG_2a_ isotype control and rabbit IgG controls (1H4L and 4H1L) were used as negative controls. **B**, **C** Flow cytometric analysis of PD-1 expression in T-cell subsets of cattle (*n* = 3). Freshly isolated bovine PBMCs were stained with anti-bovine PD-1 rabbit mAbs (1D10F1 and 4F5F2) and anti-bovine PD-1 rat mAb (5D2). Rat IgG_2a_ isotype control and rabbit IgG control (1H4L) were used as negative controls. **B** Representative histograms of staining with anti-PD-1 mAbs (orange, green, or blue histograms) and matched negative controls (gray histograms) are shown. The gating strategy of this assay was provided as Additional file 3. **C** Percentages of PD-1^+^ cells in each T-cell subset in leukocytes from healthy cattle (*n* = 3). Bars indicate the group mean percentage. Significant differences between each of the two groups were determined using a Student’s *t* test. *p < 0.05. **D** BoPD-1-Ig was preincubated with anti-bovine PD-1 rabbit mAbs (1D10F1 and 4F5F2) and anti-bovine PD-1 rat mAb (5D2), and then reacted with BoPD-L1-EGFP cells. BoPD-1-Ig bindings were evaluated by flow cytometry. Rat IgG_2a_ and rabbit IgG controls (1H4L and 4H1L) were used as negative controls. Each curve represents the relative binding of BoPD-1-Ig preincubated with each antibody compared with the no-antibody control.

Supernatants containing rabbit mAbs from two hybridoma clones were established using lymphocytes from a rabbit immunized BoPD-1-Ig and showed specific binding to BoPD-1-His protein in ELISA. These anti-bovine PD-1 rabbit mAbs were purified and tested for their binding affinity to bovine PD-1 protein by SPR analysis using a Biacore instrument. 1D10F1 and 4F5F2 showed higher affinities for PD-1 compared with 5D2, with K_D_ values of 1.11 ± 0.07, 2.85 ± 0.50, and 0.12 ± 0.04 nM, respectively (Table 1). In particular, 1D10F1 exhibited the highest association constant (ka) compared with the other mAbs (Table 1). The binding ability and specificity to bovine PD-1-expressing cells (BoPD-1-myc cells) were then tested by flow cytometry. Both anti-PD-1 rabbit mAbs (1D10F1 and 4F5F2) bound to BoPD-1-myc cells in a dose-dependent manner (Figure 1A) but not to mock cells (Additional file 2). Negative controls of rabbit mAbs (1H4L and 4H1L) did not bind to BoPD-1-myc cells at any tested concentrations (Figure 1A). 1D10F1 exhibited a stronger fluorescent intensity compared with 4F5F2 and anti-PD-1 rat mAb (5D2), which was established in our previous study (Figure 1A).

**Table 1:** **Binding affinity of anti-PD-1 mAbs to BoPD-1-His protein**

Anti-PD-1 mAb	ka (1/Ms)	kd (1/s)	KD (M)
5D2**	1.84 × 10^4^ ± 0.27	2.15 × 10^–4^ ± 0.44	1.22 × 10^–8^ ± 0.39
1D10F1	1.63 × 10^6^ ± 0.16*	1.80 × 10^–3^ ± 0.06*	1.11 × 10^–9^ ± 0.07*
4F5F2	7.39 × 10^5^ ± 0.90*	2.08 × 10^–3^ ± 0.28*	2.85 × 10^–9^ ± 0.50*

These values were measured on a Biacore X100 instrument

^*^p < 0.05 (vs. 5D2; Tukey’s test)

^**^The values for 5D2 were shown in a previous paper [10]

To determine the reactivity of anti-PD-1 rabbit mAb (1D10F1) to PD-1 naturally expressed on bovine T cells, the surface expression of PD-1 was analyzed in T-cell subsets in freshly isolated bovine leukocytes by flow cytometry. The 1D10F1 mAb recognized PD-1 expressed on CD3^+^, CD3^+^CD4^+^, CD3^+^CD8^+^, and CD3^+^γδTCR^+^ T cells, whereas 4F5F2 did not (Figure 1B). The detection sensitivity of PD-1 on the T-cell subsets by 1D10F1 was higher compared with the previously established mAb 5D2 (Figures 1B and C). Therefore, 1D10F1 has high sensitivity for the detection of bovine PD-1 in flow cytometry.

To analyze the inhibitory activity of 1D10F1 and 4F5F2, the effect of the mAbs in a binding assay with BoPD-1-Ig to BoPD-L1-EGFP expressing cells was determined by flow cytometry compared with 5D2. 1D10F1, 4F5F2 and 5D2 inhibited PD-1/PD-L1 binding in a dose-dependent manner (Figure 1D). At 50 μg/mL, the 1D10F1, 4F5F2, and 5D2 mAbs inhibited 79.2%, 54.6%, and 44.4% of PD-1/PD-L1 binding, respectively (Figure 1D). Based on these results, the mAb 1D10F1 was considered as the best blocking antibody among the established anti-bovine PD-1 mAbs.

### Establishment of an anti-bovine PD-1 rabbit–bovine chAb

**Figure 2:**
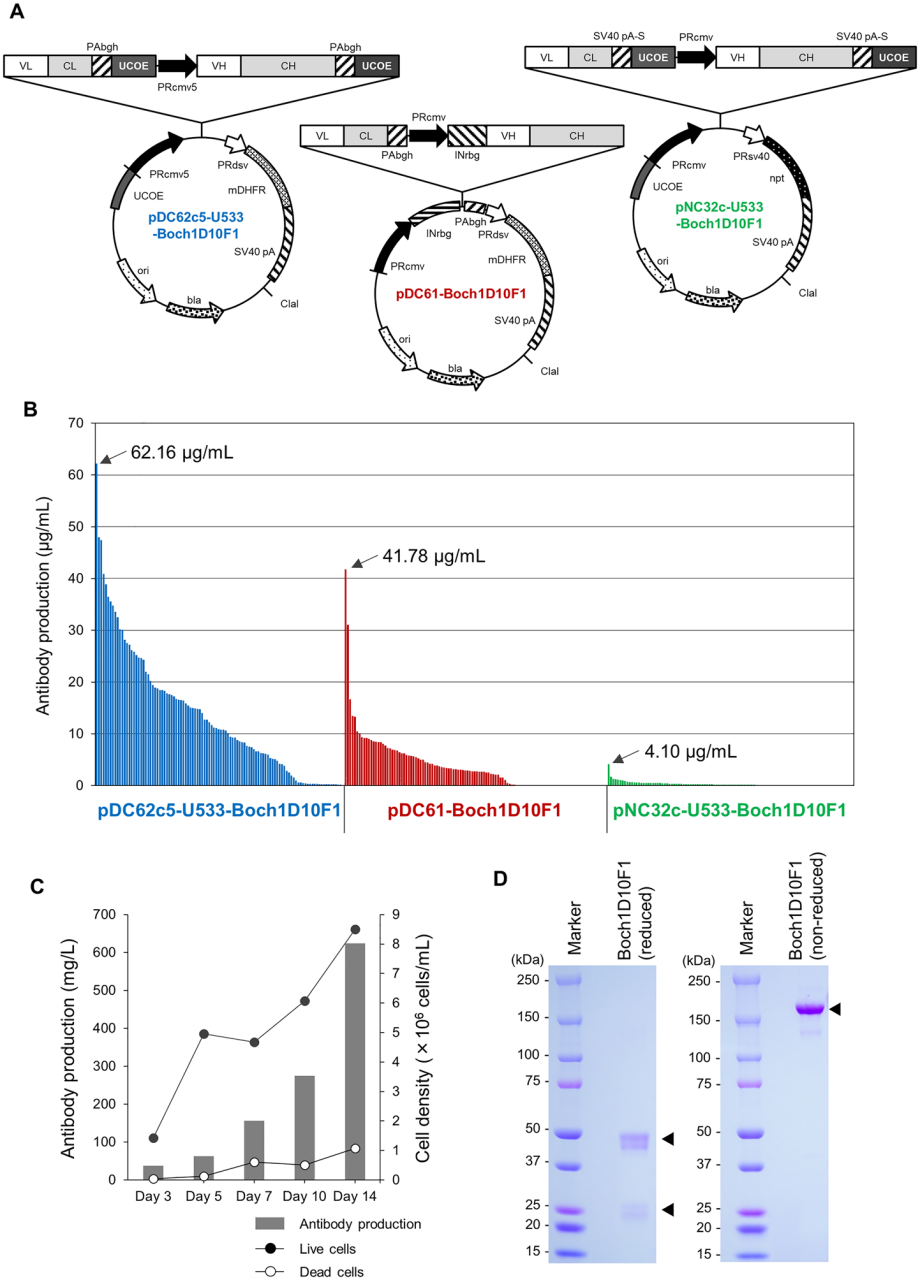
**Production of anti-PD-1 chimeric antibody, Boch1D10F1.** **A** Schematic structures of plasmid vectors encoding Boch1D10F1 (pDC62c5-U533-Boch1D10F1, pDC61-Boch1D10F1, and pNC32c-U533-Boch1D10F1). The light chain consisting of a variable region (VL) and a constant region (CL). The heavy chain consisting of a variable region (VH) and a constant region (CH). mDHFR: modified dihydrofolate reductase, UCOE: ubiquitous chromatin opening element (UCOE), npt: neomycin-resistant gene. **B** Selection of CHO DG44 cell clones producing Boch1D10F1. The production capacity of Boch1D10F1 from each cell clone transfected with pDC62c5-U533-Boch1D10F1 (blue bars), pDC61-Boch1D10F1 (red bars), and pNC32c-U533-Boch1D10F1 (green bars) are shown. The production capacity of the highest producing clones for each vector are shown in the plot. **C** Expression of Boch1D10F1. Boch1D10F1 was expressed in 2L of shaking culture of a higher-producing cell line. The antibody production (left axis: gray bar) and the density of live and dead cells (right axis: black and white circles) were measured at 3- to 4-day intervals. **D** Purification of Boch1D10F1. Boch1D10F1 was purified from the supernatants of shaking cultures. Purified protein was confirmed by reducing and nonreducing SDS-PAGE. An uncropped gel image generated during the current study is provided in Additional file 4.

The rabbit mAb 1D10F1 was engineered into a rabbit–bovine chAb, Boch1D10F1. To obtain a large amount of Boch1D10F1 for further in vitro and in vivo characterization, Boch1D10F1 was stably expressed and produced using the CHO DG44 cell expression system. Three plasmids were evaluated as an expression vector for Boch1D10F1. UCOE reduces DNA methylation in a transgene promoter and contributes to the maintenance of the expression of recombinant proteins in CHO cells [22]. pDC62c5-U533 was designed with UCOEs at the 5' and 3' ends of the expression cassettes of VL + CL and VH + CH (Figure 2A). This plasmid was modified using the pDC61 expression vector for the selection of high protein expressing cell lines using thymidine-hyposanthin regulated by the selection marker DHFR (Figure 2A), which was established previously [11, 23]. In addition, the other vector (pNC32c-U533) with UCOEs with a neomycin resistance (*npt*) selection marker was used to evaluate the performance of the combination of UCOEs and the DHFR selection marker (Figure 2A) [10, 23]. More than 100 clones were randomly selected from the cloned CHO DG44 cells transfected with each plasmid and examined for their Boch1D10F1 production by ELISA. Cells transfected with pDC62c5-U533-Boch1D10F1, pDC61-Boch1D10F1, and pNC32c-U533-Boch1D10F1 stably produced 62.2, 41.8, and 4.10 μg/mL of Boch1D10F1 after three days of shaking culture (Figure 2B). In particular, 22.6% (24/106 clones), 1.8% (2/112 clones), and 0% (0/105 clones), respectively, of the established cell lines transfected with pDC62c5-U533-Boch1D10F1, pDC61-Boch1D10F1, and pNC32c-U533-Boch1D10F1 were among the higher-producing cell lines producing > 20 μg/mL protein (Figure 2B). Thus, pDC62c5-U533 was selected for the production of recombinant antibody because high-expressing cells could be obtained efficiently.

To produce large amounts of Boch1D10F1, one of the higher Boch1D10F1-producing cell lines was cultured in 2 L of shaking culture using Dynamis Medium. Boch1D10F1 was present in the supernatants at 623.5 mg/L after 14 days (Figure 2C) and successfully purified using Protein A resin (Figure 2D). The heavy and light chains of Boch1D101 were detected at approximately 50 and 25 kDa under reducing conditions, respectively (Figure 2B). Under nonreducing conditions, Boch1D10F1 was detected as a pure, single band at > 150 kDa, which suggests that the antibody consists of two heavy and two light chains. Thus, mass production of Boch1D10F1 was achieved using a mammalian expression system.

### Characterization of anti-bovine PD-1 rabbit–bovine chAb

**Figure 3:**
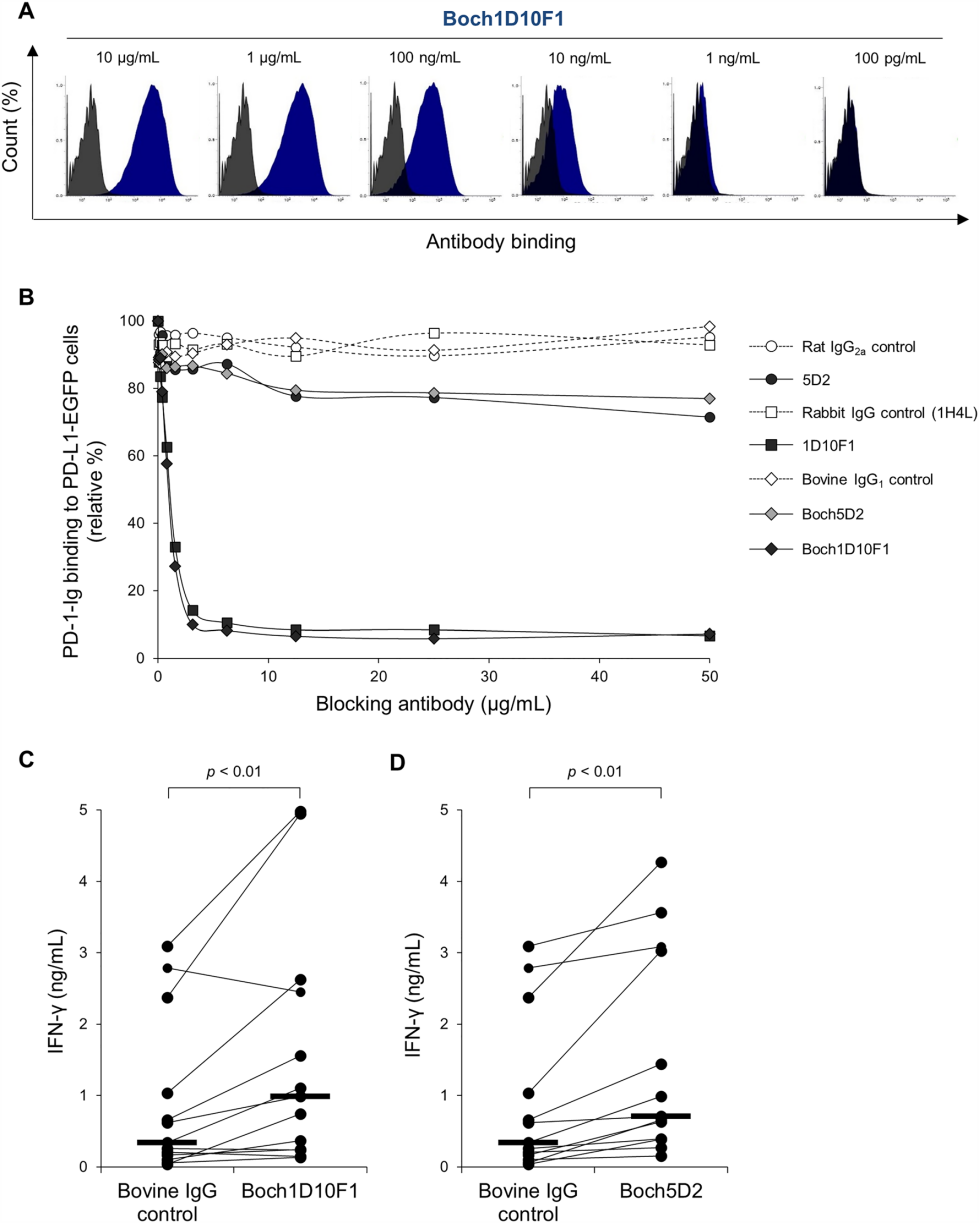
**Characterization of Boch1D10F1.** **A** Reactivity of Boch1D10F1 with BoPD-1-myc cells. BoPD-1-myc cells were stained with Boch1D10F1 in serial dilutions (10 μg/mL to 100 pg/mL) and analyzed by flow cytometry. **B** Blockade of PD-1/PD-L1 binding by Boch1D10F1. BoPD-1-Ig was preincubated with 5D2 and Boch5D2 and incubated with BoPD-L1-EGFP cells. BoPD-1-Ig bindings were evaluated by flow cytometry. Each curve represents the relative binding of BoPD-1-Ig preincubated with Boch1D10F1, Boch5D2, 1D10F1, and 5D2 compared with no-antibody control. Bovine IgG (for Boch1D10F1 and Boch5D2), rabbit IgG (1H4L), and rat IgG_2a_ controls (for 5D2) were used as negative controls. **C**, **D** Effect of PD-1/PD-L1 blockade on the IFN-γ response. PBMCs of BLV-infected cattle (*n* = 13) were cultured with Boch1D10F1 and Boch5D2 (10 μg/mL) or bovine IgG control in the presence of FLK-BLV antigen (2%) for 6 days. IFN-γ production for each animal was measured by ELISA in duplicate. Bars indicate the group median response. Significant differences between each group were determined using Wilcoxon signed-rank test Friedman test. **p < 0.01.

The binding ability of Boch1D10F1 was determined by flow cytometry using bovine PD-1-expressing cells (BoPD-1-myc cells). Boch1D10F1 was bound to BoPD-1-myc cells in a dose-dependent manner (Figure 3A). To confirm the binding affinity of Boch1D10F1 to bovine PD-1 protein, SPR analysis was performed using a Biacore 3000 instrument with BoPD-1-His protein. Boch1D10F1 and the original 1D10F1 rabbit mAb had a similar affinity for PD-1, with K_D_ values of 1.23 ± 0.05 and 1.37 ± 0.09 nM, respectively (Table 2). Therefore, the chimerization of anti-PD-1 antibody did not alter its binding affinity to bovine PD-1 protein.

**Table 2:** **Binding affinity of anti-PD-1 rabbit mAb and chAb to BoPD-1-His protein**

Anti-PD-1 antibody	ka (1/Ms)*	kd (1/s)*	KD (M)*
1D10F1	5.00 × 10^5^ ± 0.29	6.17 × 10^–4^ ± 0.38	1.23 × 10^–9^ ± 0.05
Boch1D10F1	4.37 × 10^5^ ± 0.09	6.13 × 10^–4^ ± 0.61	1.37 × 10^–9^ ± 0.09

These values were measured on a Biacore 3000 instrument

^*^The values of 1D10F1 and Boch1D10F1 are not significantly different (*p* > 0.05)

To analyze the blocking activity of Boch1D10F1, the binding of BoPD-1-Ig to BoPD-L1-EGFP-expressing cells was determined in the presence of Boch1D10F1, 1D10F1, Boch5D2, and 5D2 by flow cytometry. Boch1D10F1 inhibited the binding of PD-1/PD-L1 in a dose-dependent manner at a level similar to that of 1D10F1 (Figure 3B). In contrast, Boch5D2 and 5D2 exhibited a partial blockade of the PD-1/PD-L1 interaction (Figure 3B). Thus, Boch1D10F1 is capable of robustly inhibiting the binding of bovine PD-1/PD-L1.

The T-cell response of BLV-infected cattle is impaired by the interaction of PD-1/PD-L1 [6–8, 11]. To determine whether the treatment of Boch1D10F1 reactivates the T-cell response of BLV-infected cattle, IFN-γ production was analyzed in a cultivation assay containing PBMCs from BLV-infected cattle in the presence of Boch1D10F1 and viral antigen stimulation. The Boch1D10F1 blockade significantly enhanced IFN-γ production by PBMCs (Figure 3C), even though Boch5D2 also activated a significant IFN-γ response (Figure 3D). These results suggest that Boch1D10F1 has the potential to enhance the T-cell response in cattle.

### Reduction of proviral load by the treatment of BLV-infected cattle with Boch1D10F1

**Figure 4:**
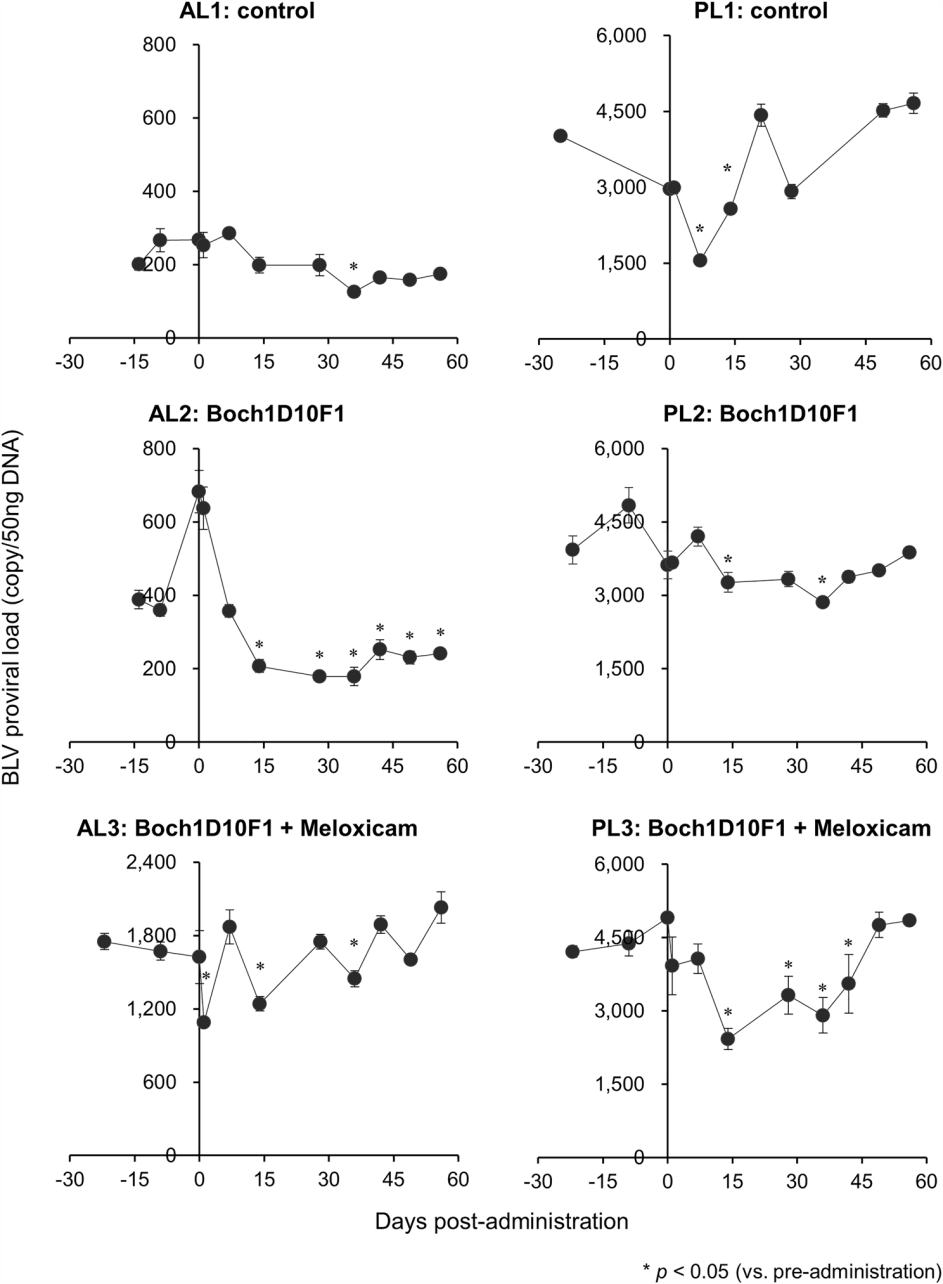
**Effect on proviral loads on BLV-infected cattle administered Boch1D10F1.** Provirus copy number per 50 ng DNA of PBMCs from BLV-infected cattle administrated Boch1D10F1 with or without meloxicam (Metacam). Proviral loads of BLV were quantified in PBMCs at each time point by real-time genomic PCR targeting the BLV *tax* gene. Each dot represents the mean of three independent experiments. Significant differences were determined by Dunnett’s multiple-comparison test across the time points. *p < 0.05 versus pre-administration. AL: aleukemic, PL: persistent lymphocytosis.

To evaluate the antiviral effect of Boch1D10F1 in vivo, BLV-infected cattle were inoculated with anti-PD-1 chAb (Boch1D10F1). AL2 and PL2 were treated with Boch1D10F1 intravenously at a dose of 1 or 2 mg/kg, respectively. AL3 and PL3 also received an intravenous dose of Boch1D10F1 (1 or 2 mg/kg) on day 0 as well as a subcutaneous injection of 0.5 mg/kg meloxicam (Metacam) on day 0, 7, and 14, because the combination of PD-1/PD-L1 blockade and COX-2 inhibition represents a promising strategy to synergistically enhance the antiviral T-cell response during BLV infection [12, 30]. Among the treated animals, AL2 and PL3 exhibited a sustained decrease in BLV proviral load in PBMCs following Boch1D10F1 administration (Figure 4).

## Discussion

In this study, two high-affinity rabbit mAbs (1D10F1 and 4F5F2) against bovine PD-1 were successfully generated. The mAb 1D10F1 was very useful for the detection of bovine PD-1 in immunostaining for flow cytometry and exhibited strong inhibition of the PD-1/PD-L1 interaction, compared with the rat mAb 5D2. In contrast, 4F5F2 was also expected to be a good detection antibody because of its high binding affinity to the extracellular region of the PD-1 soluble protein (BoPD-1-His) in the Biacore assay (Table 1), but flow cytometric assays with this mAb detected a smaller percentage of full-length PD-1 protein in BoPD-1-myc overexpressing cells (Figure 1A) and none at all in bovine T cells (Figure 1B). This is presumably because the epitope to which 4F5F2 binds could be a motif not exposed on the surface of full-length PD-1 protein expressed at plasma membrane. More validation is needed to explain well why the seemingly contradictory results were obtained for 4F5F2.

We have been working on the establishment and characterization of bovine chAb [10, 11, 31], and this study represents our first attempt at establishing a rabbit–bovine chAb to achieve increased binding affinity to an antigen. The anti-PD-1 rabbit–bovine chAb (Boch1D10F1) was generated in a high-producing cell line selected from more than 300 cell clones. As reported previously, the addition of UCOE into the expression plasmid also increased the expression level of the transgene compared with conventional plasmids in our experiments. Although we have not examined the mechanism of this increase, UCOE is known to inhibit DNA methylation in the transgene promoter and maintain the expression level of the transgene after many passages of the established cell line [22, 23]. In the present study, Boch1D10F1 was prepared for animal experiments using adult cattle in a flask-shaking culture system. We established as system to develop a higher antibody producing cell line, which will be very useful for in vivo antibody research using large animal models, such as cattle, dogs, pigs, horses, and monkeys.

Although Boch1D10F1 exhibited a higher binding affinity and stronger blocking activity to bovine PD-1 compared with Boch5D2 [10], immune activating efficacy was observed in PBMC cultivation assays using these chAbs. IFN-γ was measured as a representative cytokine as well as a key regulator of Th1 response based on our previous studies [7, 8, 10]. We attempted to measure other cytokines, such as IL-2 and TNF-α using ELISA, but they were not detectable in the culture supernatant. To determine the blockade effect in EBL animals, other methods to detect cytokine production from BLV-specific T cells are needed, such as the Enzyme-Linked ImmunoSpot Assay or Luminex Assay System with immunogenic peptides or proteins [32–34].

With the spread of BLV on farms throughout Japan [35], the incidence of EBL has increased in recent years, causing significant economic losses for dairy and beef farmers. Because of the high prevalence in Japan, it has been extremely difficult to implement cleanup through the detection and culling of infected cattle. Furthermore, no effective treatment or vaccine has become commercially available at this time. Therefore, a novel control strategy against BLV infection is required.

In the animal experiment of Boch1D10F1 administration, PD-1 blockade reduced the BLV proviral load in one of the aleukemic (AL) cattle (AL2), which is consistent with a previous observation in a pilot clinical trial of Boch5D2 [10]. The antiviral effect in this animal was observed throughout the experiment. In contrast, a continuous antiviral effect was not observed in PL2, which is also consistent with our previous study demonstrating that PD-1/PD-L1 and COX-2/prostaglandin E2 (PGE_2_) pathways cooperatively suppresses the T-cell response in BLV-infected cattle with progressive disease, such as persistent lymphocytosis (PL) animals [12]. Consistently, the combination of Boch1D10F1 and meloxicam induced an antiviral effect in a PL cow (PL3); however, the viral load was reversed at day 49 post-administration. The mechanism of this increase may be a decreased blocking effect resulting from antibody degradation or compensation by other immunosuppressive factors, such as the immune checkpoint molecule, cytotoxic T-lymphocyte antigen 4 (CTLA-4), or regulator T-cell subsets [36–38]. These possibilities should be tested in subsequent clinical studies through repeated administration of chAb or a combination with other blocking antibodies, such as anti-CTLA-4 chA﻿b [31]. In addition, phenotyping T-cell subsets in these animals will be important to understand the varying efficacy of PD-1 blockade observed in each individual.

We demonstrated that treatment with anti-PD-L1 chAb activated the T-cell response in cattle infected with *Mycobacterium avium* subsp. *paratuberculosis* and *Mycoplasma bovis* [39–41]. The clinical benefit of Boch1D10F1 treatment may also be determined in cattle with paratuberculosis and mycoplasmosis anaplasmosis to determine whether targeting PD-1/PD-L1 pathway can be applied as broad-spectrum immunotherapy against chronic infectious diseases in cattle. In addition, functional role of PD-1/PD-L1 pathway in T-cell response of other bovine infections should be clarified, such as bovine tuberculosis, brucellosis, babesiosis, and theileriosis to consider the spectrum of this strategy. Furthermore, bovinized antibodies, in which the frameworks of the variable region are replaced with bovine antibody, are expected to improve stability in vivo and further enhance clinical efficacy and could be the next option of the strategy for bovine immunotherapy.

To develop new control methods for chronic infectious diseases, it is necessary to further analyze the immunosuppressive mechanisms of each disease and to consider the most effective combination of therapies (therapeutic targets). In addition, the development and production costs of antibody drugs must be fully considered. This study provides a technological basis for producing high performance antibodies in large quantities and at low cost.

### Supplementary Information

**Additional file 1. Animals used in the experiment of antibody administration.** Six BLV-infected cattle (Holstein, female) were used for the administration experiments of Boch1D10F1 and meloxicam to evaluate their antiviral effects in vivo.

**Additional file 2. Flow cytometric analysis using anti-bovine PD-1 mAbs to mock cells.** Flow cytometric analysis was performed using anti-bovine PD-1 rabbit mAbs (1D10F1 and 4F5F2) and anti-bovine PD-1 rat mAb (5D2) and mock-transfected CHO DG44 cells. CHO DG44 cells were transfected with pCI-neo (mock plasmid) and cloned by limiting dilution. The mock cell line was stained with the mAbs (100 μg/mL) at room temperature for 30 min. Rat IgG_2a_ isotype control and rabbit IgG controls (1H4L and 4H1L) were used as negative controls. The cells were labeled with Alexa Flour 647-conjugated anti-rabbit IgG (H + L) goat F(ab')2 (Thermo Fisher Scientific), APC-conjugated anti-rat immunoglobulin antibody (Southern Biotech) at room temperature for 30 min. Finally, the cells were washed and analyzed immediately using FACS Verse (BD Biosciences).

**Additional file 3. Gating strategy of T-cell subsets in leukocytes from healthy cattle.** CD4^+^, CD8^+^, or γδTCR^+^ T cells were gated in CD3^+^IgM^−^ lymphocytes and then analyzed for expression of PD-1 in each T cell subset as shown in Figure 1B.

**Additional file 4. An uncropped gel image for Figure ****2****D.** Purified anti-PD-1 chAb (Boch1D10F1) was analyzed by reducing and nonreducing SDS-PAGE.

## Data Availability

The datasets used during the current study are available from the corresponding author on reasonable request.
