# Supplementary figures and images for: Development of a high-affinity anti-bovine PD-1 rabbit–bovine chimeric antibody using an efficient selection and large production system
Source: Vet Res. 2023 Sep 27;54:82. doi: 10.1186/s13567-023-01213-6 (PMC10537840; doi:10.1186/s13567-023-01213-6)

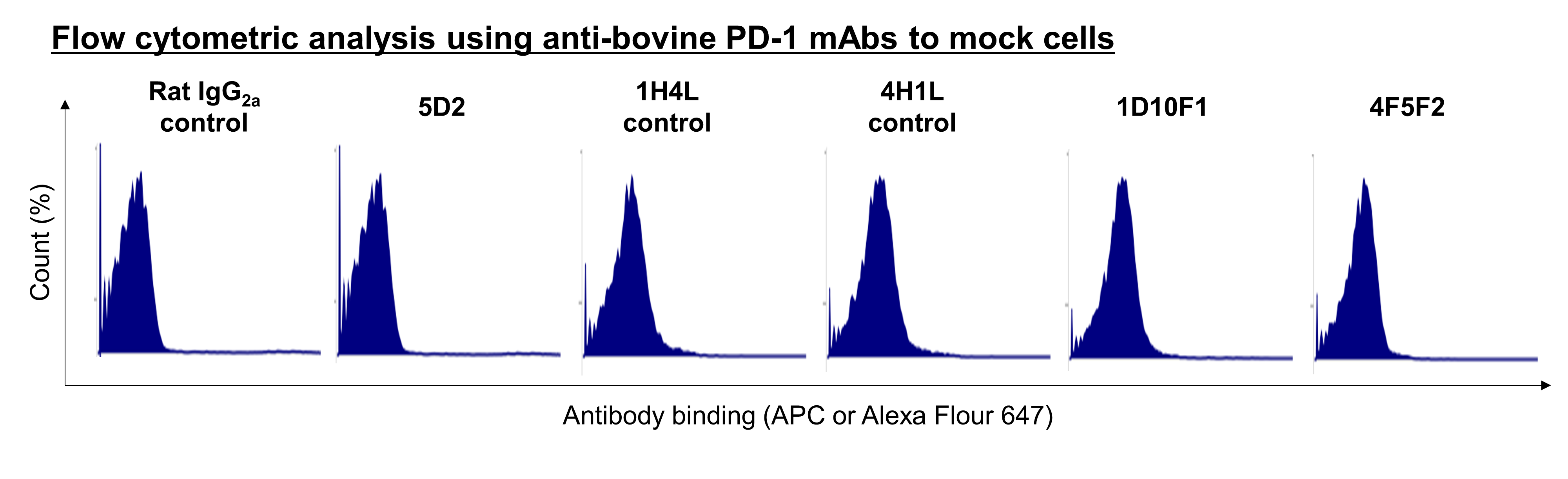

Supplement: Supplementary file 2 — Additional file 2. Flow cytometric analysis using anti-bovine PD-1 mAbs to mock cells. Flow cytometric analysis was performed using anti-bovine PD-1 rabbit mAbs (1D10F1 and 4F5F2) and anti-bovine PD-1 rat mAb (5D2) and mock-transfected CHO DG44 cells. CHO DG44 cells were transfected with pCI-neo (mock plasmid) and cloned by limiting dilution. The mock cell line was stained with the mAbs (100 μg/mL) at room temperature for 30 min. Rat IgG2a isotype control and rabbit IgG controls (1H4L and 4H1L) were used as negative controls. The cells were labeled with Alexa Flour 647-conjugated anti-rabbit IgG (H + L) goat F(ab')2 (Thermo Fisher Scientific), APC-conjugated anti-rat immunoglobulin antibody (Southern Biotech) at room temperature for 30 min. Finally, the cells were washed and analyzed immediately using FACS Verse (BD Biosciences). [file 13567_2023_1213_MOESM2_ESM.tif]

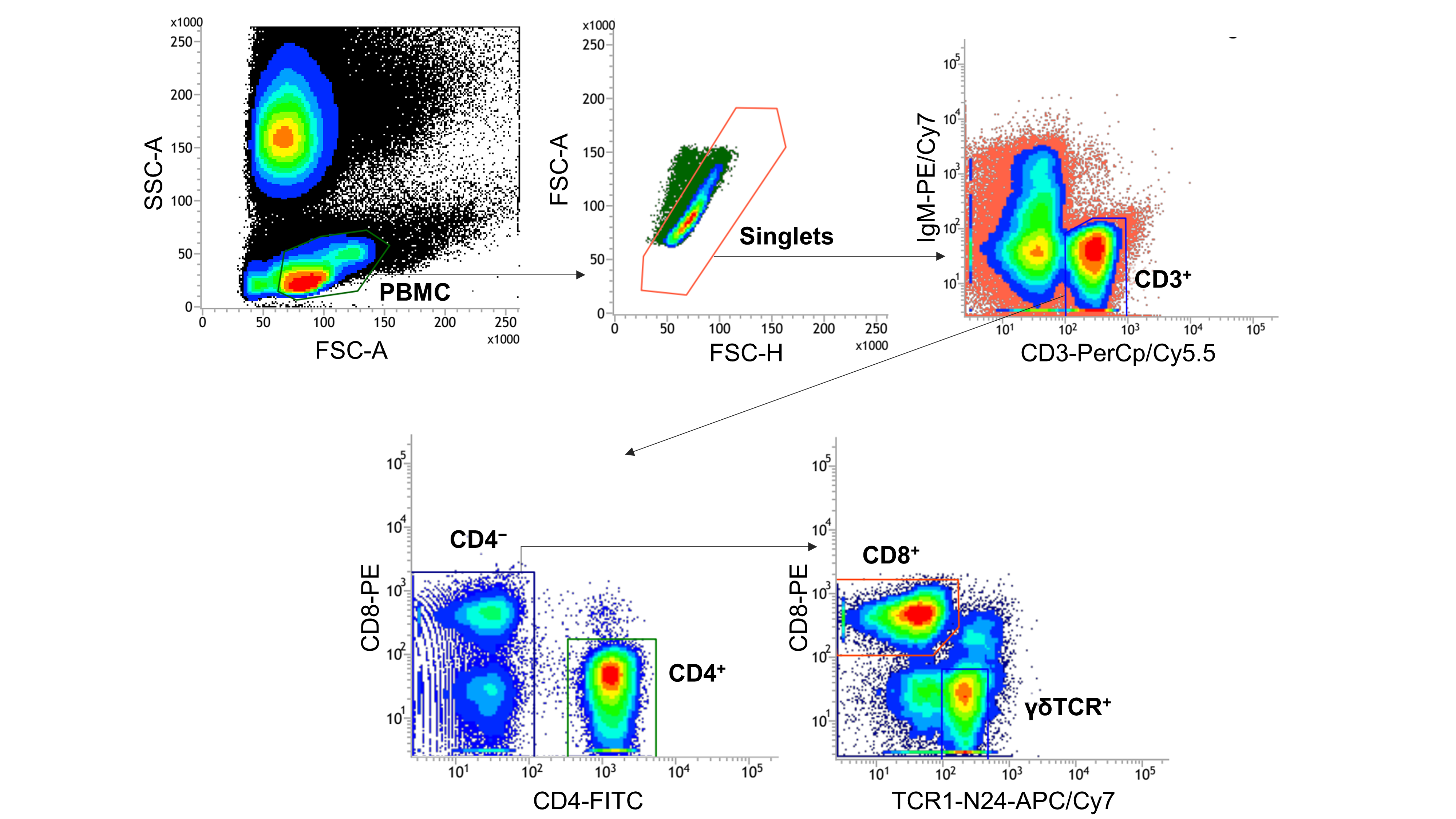

Supplement: Supplementary file 3 — Additional file 3. Gating strategy of T-cell subsets in leukocytes from healthy cattle. CD4+, CD8+, or γδTCR+ T cells were gated in CD3+IgM− lymphocytes and then analyzed for expression of PD-1 in each T cell subset as shown in Figure 1B. [file 13567_2023_1213_MOESM3_ESM.tif]

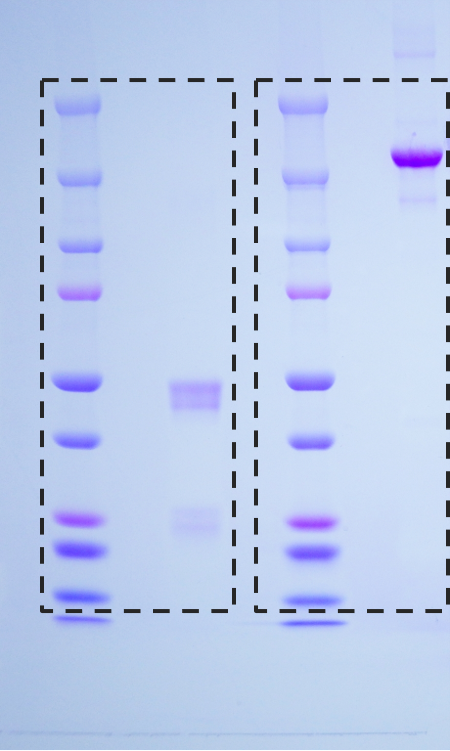

Supplement: Supplementary file 4 — Additional file 4. An uncropped gel image for Figure 2D. Purified anti-PD-1 chAb (Boch1D10F1) was analyzed by reducing and nonreducing SDS-PAGE. [file 13567_2023_1213_MOESM4_ESM.tif]
